# Lectures on the Theory and Practice of Surgery

**Published:** 1846-01

**Authors:** 


### ART. XXIII.

*Lectures on the Theory and Practice of Surgery.* By the late ABRAHAM COLLES, M.D., &c. Edited by SIMON M'COY, Esq., F.R.C.S.I.—*Dublin and London,* 1845. 2 vols. 8vo, pp. 396-360.

THESE Lectures, by the late respected Professor of Surgery in the Royal College of Surgeons in Ireland, have been reprinted from the 'Dublin Medical Press,' and put into their present more commodious form by Mr. M'Coy, who had the advantage of a personal attendance during their delivery. They have "been compiled from notes of several courses, and carefully collated with the manuscripts of others of Mr. Colles's pupils." They are indeed "eminently practical," and we doubt not must have had great influence in their time. There is no effort at what might be called scientific arrangement, and this we take as proof of Mr. Colles's practical character, for such an attempt would only have involved theoretical disquisition—a thing which seems to have been most carefully avoided by the teacher. The topic of each lecture is clearly stated in simple and unaffected language; the illustrative cases, anecdotes, and occasional witticisms, are all much to the point; and, even without the precious impress of Mr. Colles's name, any practical man looking over these pages would at once perceive that he was reading the doctrines of a master in the art.

We imagine, however, that the chief value of these lectures is derived from the circumstance that they were those of Abraham Colles. We doubt not that they will be received with pleasure by many in Ireland, who cherish with respect and gratitude the memory of one who guided them so judiciously in the early part of their career. Further than this we do not suppose that they will be accepted; and though we can most conscientiously recommend them as being in every way worthy of consideration as far as they go, we feel equally bound as impartial critics to state, that they are far behind the surgery of the present day. While doctrines, many of which may in a manner be deemed obsolete, are thus published, there is not a word about the modern improvements in surgery. There is not a sentence from Mr. Colles about lithotrity, for example; nor of the hundred little matters of novelty introduced within the last twenty years. Mr. M'Coy, to be sure, gives a foot-note about lithotrity, whereby it might appear, to a person not acquainted with the state of surgery, that this proceeding had actually been alluded to, even in a manner exhibited, in a lecture-room in Dublin. We are gravely told by Mr. M'Coy that "at this part of the lecture, and before the operation (lithotomy) was demonstrated in the dead body, by Mr. Colles, an Italian surgeon, named, I think, Pacheoli, was introduced to the class, one session, to explain the mechanism and *modus operandi* of an instrument he contrived for lithotrity;"— and so the note proceeds, in a like innocent tone, to the end, without one word of what has been done since. Surely Mr. Colles must have taken more notice of such a subject in subsequent courses of his lectures! If so, as "the present publication has been compiled from notes of several courses," it is remarkable that we have no further account of it.

Both teacher and annotator occasionally display a degree of simplicity which makes us smile. Thus we are told in the lecture on hydrophobia, (p. 89,) that Mr. Colles has "been informed that spring is the time dogs are most subject to madness in Scotland, and it is accounted for in this way. A number of cattle are annually left to perish during the severity of the winter, and it is supposed the dogs over-eat themselves, which causes a tendency to the disease," and the worthy professor sagely concludes, "but whether this be correct or not, I cannot say." There was no Rowland Hill in those days, yet "we have been informed" that there was a post between Ireland and Scotland, and imagine that a letter would—after a week or two—have put an end to all doubt in the mind of the worthy teacher.

The omission of many characteristics of modern surgery, however, cannot be deemed a fault in these lectures—for such things were not known when Mr. Colles taught. "The medical practitioners of Ireland of the present day," to whom the preface of the publisher is specially addressed, must not, therefore, expect from these volumes an exposition of the present state of surgery; they must take them, as we have already said, only as Mr. Colles's lectures.

There are only forty-nine lectures in all, and it would therefore be unreasonable to expect that Mr. Colles should have discussed every subject of surgery. We cannot but express our astonishment, however, that out of this small number ten of them should be devoted to the venereal disease. But the subject has seemingly always been a favorite one with the Dublin surgeons.

